# The Insomnia Severity Index: Factor Structure and Measurement and Structural Invariance across Perinatal Time Points

**DOI:** 10.3390/healthcare11081194

**Published:** 2023-04-21

**Authors:** Eriko Shinohara, Ayako Hada, Mariko Minatani, Mikiyo Wakamatsu, Toshinori Kitamura

**Affiliations:** 1 Department of Nursing, School of Medicine, Yokohama City University, Yokohama 236-0004, Japan; 2 Kitamura Institute of Mental Health Tokyo, Tokyo 151-0063, Japan; 3 Kitamura KOKORO Clinic Mental Health, Tokyo 151-3306, Japan; 4 Department of Community Mental Health & Law, National Institute of Mental Health, National Center of Neurology and Psychiatry, Tokyo 187-8553, Japan; 5 Life Value Creation Unit, NTT DATA Institute of Management Consulting, Inc., Tokyo 102-0093, Japan; 6 Department of Reproductive Health Care Nursing, Kagoshima University Faculty of Medicine School of Health Sciences, Kagoshima 890-8544, Japan; 7 T. and F. Kitamura Foundation for Studies and Skill Advancement in Mental Health, Tokyo 151-0063, Japan; 8 Department of Psychiatry, Graduate School of Medicine, Nagoya University, Nagoya 466-8550, Japan

**Keywords:** insomnia severity index, factor structure, measurement and structural invariance, parity, pregnant women, pregnancy

## Abstract

Insomnia is associated with adverse outcomes in women in the perinatal period; thus, the assessment of insomnia is important for pregnant women. The Insomnia Severity Index (ISI) is an instrument used globally to assess the severity of insomnia. However, its factor structure and structural invariance for pregnant women have not been studied. Therefore, we aimed to conduct factor analyses to search for the best model to fit its structural invariance. A cross-sectional study with the ISI was conducted at one hospital and five clinics in Japan from January 2017 to May 2019. A set of questionnaires was administered on two occasions with a one-week interval. The study included 382 pregnant women ranging in gestational age from 10 to 13 weeks. One week later, 129 participants answered the retest. After exploratory and confirmatory factor analyses, the measurement and structural invariance between parity and two time points was tested. The two-factor structure model showed an acceptable fit for the ISI in pregnant women (*χ*^2^ (12) = 28.516, CFI = 0.971, RMSEA = 0.089). The model also showed satisfactory measurement and structure invariance between parity and time points. The findings indicate that the ISI’s use would be appropriate for pregnant women as a two-factor subscale of “severity” and “impact”, regardless of the parity or time point. The ISI’s factor structure may vary by subject; hence, it is necessary to confirm the measurement and structural invariance of the subject for whom the ISI will be used. Furthermore, interventions that focus not only on total scores and cutoff points but also on the phenomenon of subscales should be considered.

## 1. Introduction

Insomnia is a relatively common health disorder experienced by a large number of people, with approximately 10% of the adult population suffering from insomnia and an additional 20% experiencing occasional insomnia symptoms [1]. As for the risk, women, the elderly, and those with socioeconomic difficulties are more likely to suffer from insomnia [1]. Insomnia symptoms includes delayed onset, continuation, or poor quality of sleep with functional impairments. The DSM-5 diagnostic criteria for insomnia disorder, the leading international standard, lists the presence of significant complaints of dissatisfaction with the quantity or quality of sleep for insomnia [2]. The criteria include one or more of the following symptoms: difficulty falling asleep, frequent awakenings, difficulty staying asleep, and difficulty falling back to sleep due to early morning awakenings [2]. They are also listed as causing dysfunction, occurring at least three nights a week, occurring for three months, and occurring despite adequate sleep opportunities, and not explained by other sleep-wake disorders, substance abuse, or co-existing psychiatric or medical illness [2]. Pregnant women are likely to experience sleep disorders because of hormonal or psychological changes [3,4]. In early pregnancy, women are prone to daytime drowsiness induced by progesterone. Morning sickness, minor problems such as frequent urination and back pain due to an enlarged uterus, fetal movement, and anxiety about childbirth also affect sleep quality [5]. The characteristics of sleep in pregnant women, compared with non-pregnant women, include a greater number of awakenings after falling asleep, less REM sleep, and more time spent in the lighter sleep stages [6,7]. Regarding the rate of sleep disturbances in pregnant women, it depends on the scale used and the timing. The prevalence of sleep problems increases in later terms [5], and most women experience nocturnal awakenings in the third trimester [5]. With respect to sleep quality, about half of all pregnant women report poor sleep quality [8], and the percentage of pregnant women with clinically severe insomnia symptoms as measured by the Insomnia Severity Index (ISI) has been reported to range over 14–27% [9,10]. Poor sleep quality may be linked to adverse perinatal events, such as preterm birth [11,12] or prolonged labor leading to caesarean section [13], as well as psychological disorders, such as increased depressive symptoms or anxiety during pregnancy and postpartum [14,15].

Since primary anxiety disorders such as major depressive disorder, bipolar, generalized anxiety disorder, post-traumatic stress disorder, panic disorder, and obsessive–compulsive disorder can manifest as pregnancy-related insomnia, a careful differential diagnosis is necessary [5]. The differential diagnosis of mood disorders, breathing-related sleep disorders, and restless legs syndrome is also needed [5]. There are several methods to assess sleep, including polysomnography, sleep diaries, and self-assessment forms using questionnaires. The evaluation of insomnia with reliable and valid instruments is essential for diagnosis and intervention [16]. There are several insomnia screening questionnaires, such as the Pittsburgh Sleep Quality Index (PSQI) [17], Insomnia Symptom Questionnaire [18], Athens Insomnia Index [19], and ISI [20]. Among them, the PSQI is often used as a screening tool for sleep disorders because it is useful and easy to administer; however, it primarily assesses sleep quality and does not fully meet the diagnostic criteria for insomnia or target the degree of disability or mental distress associated with insomnia [20]. In contrast, the ISI is a brief and valid questionnaire used in clinical situations that covers the subjective symptoms and consequences of insomnia and the degree of worry and distress caused by these difficulties; further, its content corresponds partly to the diagnostic criteria for insomnia [20]. Used as a DSM-5 criterion for insomnia, the ISI is an excellent rating index for detecting insomnia in pregnant women [21].

The ISI has been translated into several languages and its factor structure has been studied in various populations worldwide. It has been found that the ISI’s factor structure differs among populations with different features. For patients with sleep disorders, two [22] and three factors [20,23] have been reported. For chronic pain patients, a one-factor solution in an exploratory factor analysis (EFA) was confirmed, but this model did not fit in the confirmatory factor analysis (CFA); therefore, a shorter version of the ISI was developed [24]. One study, with a target population of school-aged children, confirmed a two-factor structure [25]. For adolescents, a one-factor model was excellent [26]. For menopausal participants, a two-factor structure was confirmed [27]. In cross-country research on general adult populations and in Spain, a three-factor structure was confirmed [28,29]. For pregnant women, the PSQI is more frequently used as an insomnia scale, and the ISI has been used in few studies among perinatal populations [9,10]; the factor structure of the ISI for pregnant women has not been studied yet.

In addition to the factor structure, confirming configural invariance, measurement invariance, and factor invariance in the use of measurement scales is an important research topic [30]. This is because if the factor structure of a scale (configural invariance), the factor loadings of an indicator or item (metric invariance: weak measurement invariance), the intercept of an indicator (scalar invariance: strong measurement invariance), and the residual of an indicator (residual invariance: strict measurement invariance) are significantly different among people with different backgrounds, the validity of the scale will be questioned. There are three types of measurement invariance: metric invariance, scalar invariance, and residual invariance. The factor variance, factor covariance, and factor mean of a psychological scale must be equivalent among people with different backgrounds if used as a means of comparison [31].

Currently, only Chen et al. [28] have explored confirmed the measurement invariance of the ISI for the general adult population between countries. The factor structures may differ based on the target population. Even within the same target population, the reliability of the scale is not assured if the factor structures of the characteristics (e.g., primipara and multipara) and timing (e.g., gestational weeks) are different. To confirm the equivalent structure of the scale within the target population, an analysis of measurement and structure invariance is essential. For example, even if the target population is considered the same group, they may be measured differently due to subtle differences in their characteristics. Therefore, we aimed to examine the measurement and structure invariance of the ISI targeted toward pregnant women without bias of parity and time.

## 2. Materials and Methods

### 2.1. Procedures and Participants

This cross-sectional observational study was conducted in antenatal clinics of one general hospital and five private clinics located in Tokyo, Chiba, Ibaraki, and Kagoshima prefectures in Japan, from January 2017 to May 2019.

**Figure 1:**
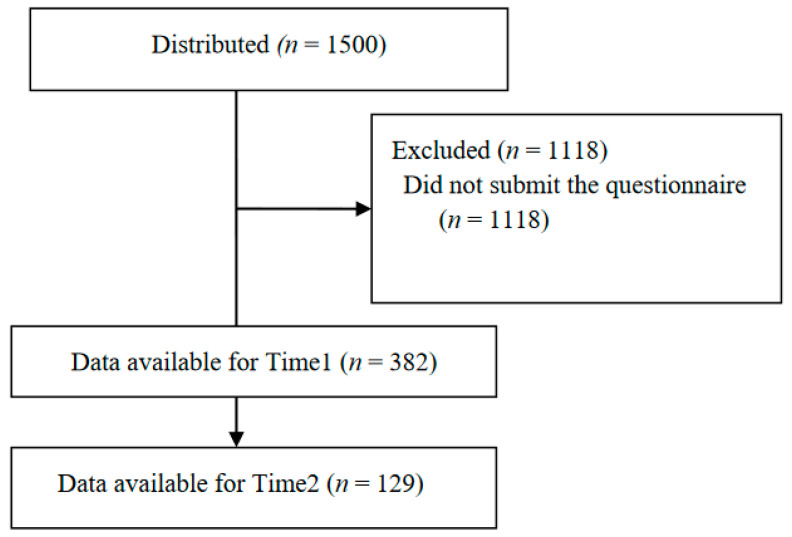
A Study Flowchart.

The participants were pregnant women at 10 to 13 weeks of gestation because hyperemesis and sleep were investigated. We excluded pregnant women who were illiterate in Japanese; under 20 years of age; had eating disorders, symptoms of vaginal bleeding or abdominal pain, or subchorionic hematoma; and were diagnosed with recurrent miscarriages. The questionnaire was distributed to 1500 women via research assistants at study facilities. A set of questionnaires was administered on two occasions with a one-week interval (times 1 and 2). A total of 382 (approximately 25%) pregnant women responded to the questionnaire survey. Among them, 129 responded for the retest (Time 2) (Figure 1). The responses at both time points were matched by a predetermined number on the questionnaires.

The mean ages (SD) of the participants and their partners were 31.9 (4.9) and 33.5 (5.5) years, respectively, and 94% were married. More than half (55%) of the participants were multiparas and 44% were nulliparas. Regarding their educational background, 43% were university graduates and 36% were college graduates. The mean household income was 6,780,000 yen (49,370 US dollars). In terms of their working status, more than half (56%) were employed, 10% were on parental leave, and 34% were housewives.

### 2.2. Measurements

The ISI is a self-rating questionnaire that assesses the nature, severity, and impact of insomnia in the past two weeks [16,20]. The ISI contains seven items with a five-point Likert type scale (0 = “no problem” to 4 = “very severe problem”), with the total scores ranging from 0 to 28, whereby a higher score indicates severe insomnia. The Japanese version was translated by Munezawa et al. [32]. A cutoff value of 10 is considered reasonable for insomniacs and normal subjects [20], but some studies have used 10 or 12 for Japanese subjects [32,33].

### 2.3. Data Analysis

#### 2.3.1. EFA and CFA

The datasets were split into two: one for the EFA (*n* = 184) and another for the CFA (*n* = 198). Before performing the EFA, excessive skewness and kurtosis (>2.0) for each item and extremely low communality (>0.02) were examined to check the normal distribution of data of each item. Little’s Missing Completely at Random (MCAR) test showed that the data were missing completely at random: *χ*^2^ (*df*) = 12.655 (11) (*p* = 0.316). Therefore, we treated missing values with listwise deletion. The Kaiser–Meyer–Olkin (KMO) index and Bartlett’s sphericity test were used for the factorability of the items. The EFA was performed using the maximum likelihood method with PROMAX rotation to develop an appropriate model. Next, using the other half of the data, the CFA was conducted to confirm the best-fit model. To assess the fit of the models, three different indices were used: *χ*^2^, the comparative fit index (CFI), and root mean square of error approximation (RMSEA). A good fit was defined as *χ*^2^/*df* < 2, CFI > 0.97, and RMSEA < 0.05. An acceptable fit was defined as *χ*^2^*/df* < 3, CFI > 0.95, and RMSEA < 0.08 [34].

#### 2.3.2. Measurement Invariance and Structure Invariance

After confirming the best-fitting model by performing the EFA and CFA, we assessed its measurement and structure invariance for primipara vs. multipara and time 1 vs. time 2. Four individuals whose parity was unknown were excluded from the analysis. Referencing Vandenberg and Lance [31], we used seven steps to confirm the measurement and structure invariance: (a) configural invariance: each group (i.e., nulliparas and primiparas and time 1 and time 2) has the same pattern of indicators and factors; (b) metric invariance: factor loadings for similar indicators are invariant across groups; (c) scalar invariance: intercepts of similar items are invariant across groups; (d) residual invariance: residuals of similar items are invariant across groups; (e) factor variance invariance: variances of similar factors are invariant across groups; (f) factor covariance invariance: covariances between factors are invariant across groups; and (g) factor mean invariance: factors are invariant across groups. We stepped up the process if there was a non-significant increase in *χ*^2^ for the *df* or difference, a decrease in the CFI by less than 0.01, or an increase in RMSEA by less than 0.01 [34,35]. All data were analyzed using IBM SPSS Statistics and Amos for Windows version 24.0 (IBM Co., Ltd., Tokyo, Japan).

### 2.4. Ethical Considerations

This study was approved by the ethical committee of the Kitamura Institute of Mental Health, Tokyo (No. 2015052301), and Kagoshima University (No. 170247).

## 3. Results

### 3.1. EFAs

The mean, *SD*, skewness, and kurtosis of the ISI items are presented in Table 1. The skewness and kurtosis of most ISI items were low. Thus, normality of the data distribution was assumed (KMO = 0.854, Bartlett’s sphericity *χ*^2^ (21) = 642.635 (*p* < 0.001), and communality of all items > 0.40). The factorability was adequate; therefore, we performed EFAs with PROMAX rotation next using the maximum likelihood method from single-, two-, and three-factor structures (Table 2). However, in the three-factor model, only one item (item 4) loaded on the third factor with 0.3 or more.

### 3.2. CFAs, Measurement Invariance, and Structure Invariance

**Figure 2:**
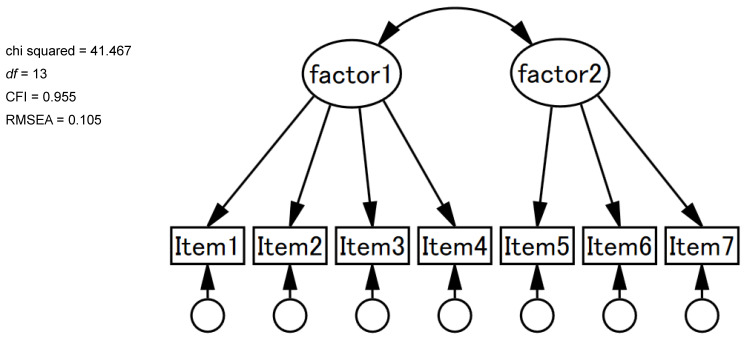
Two-factor model of the ISI for pregnant women (*n* = 198).

The one-factor structure model was not an acceptable fit with the data (*χ*^2^ (14) = 88.997, *χ*^2^/*df* = 6.357, CFI = 0.881, RMSEA = 0.165); however, the two-factor structure model showed an acceptable fit (*χ*^2^ (13) = 41.467, *χ*^2^/*df* = 3.190, CFI = 0.955, RMSEA = 105) (Figure 2). Therefore, the two-factor structure model was selected as the best model. The items comprising factor 1 were the severity of sleep onset, sleep maintenance, early morning awakening problems, and sleep satisfaction. Factor 2 included interference of sleep difficulties with daytime functioning, noticeability of sleep problems by others, and distress caused by sleep difficulties. Based on the content of the items, factor 1 was related to the severity of sleep symptoms and factor 2 was related to the impact on daytime activities. Therefore, we named factor 1 “severity” and factor 2 “impact.”

Then, we examined the measurement and structure invariance between nulliparas and multiparas and times 1 and 2. The two-factor model for the ISI was invariant with configural, metric, scalar, factor variance, and factor covariance perspectives for both parity and time points (Table 3). The factor means for both the severity and impact were not significantly different across parity and time points (Table 4).

## 4. Discussion

To the best of our knowledge, this is the first study to examine the ISI’s factor structure and measurement and structural invariance across perinatal time points among pregnant women. This study showed that a two-factor model (severity and impact) for the ISI can be used for pregnant women regardless of the parity or time points. Its measurement and structural invariances were confirmed across parity points and a week-apart time measurement among women in the first trimester.

Although approximately 20 studies have been published to date on the ISI factor structure, only one-quarter of them have conducted both an EFA and CFA [36]. Previous systematic reviews and meta-analyses of the structural validity of the ISI have shown that a two-factor solution was a more robust presentation of dimensionality compared to a three-factor solution [36]. Our results presented a two-factor model consisting of “severity” (with four items) and “impact” (with three items) components. Items 1–3 originally included questions about severity, such as the severity of sleep onset, sleep maintenance, and early morning awakening problems, and item 4 explored sleep satisfaction based on subjectivity, which reflects the “severity”. Items 5 and 6 were originally about daytime quality of life and item 7 focused on the distress caused by sleep difficulties. Therefore, three items reflected the “impact”. Each factor is considered reasonable in measuring aspects of an individual’s actual sleep. Focusing on constructs, only targeting menopausal women in a previous study was the same construct of the ISI [27]. Several previous studies used the same item construct subscale as factor 2 in our model (i.e., interference of sleep difficulties with daytime functioning, noticeability of sleep problems by others, and distress caused by sleep difficulties), which was named “impact” [23,28,29]. Although most previous studies using the ISI have been conducted on mixed-sex participants, the factor structures may differ owing to sex-based differences in sleep characteristics or due to individuals and research on subscales of the scale for the subject to be used.

Most women experience sleep disturbances during pregnancy, including frequent awakenings and difficulty falling asleep [37]. In addition, the characteristic hormonal changes caused by physical changes during pregnancy, morning sickness, frequent urination, and fetal symptoms can easily lead to poor sleep quality [5]. The characteristics of sleep disorders in pregnant women are unique from those in the general adult population, and it is important to use a specific index to diagnose sleep disorders in pregnant women. For the evaluation of sleep disorders, the cutoff points for being considered a sleep disorder in pregnant women are ISI ≥ 10 and ISI ≥ 11 when compared to the DSM-5 criteria [21]. On the ISI, higher scores indicate more severe insomnia [20]. While it is important to diagnose sleep disturbances using the cutoff values or severity for pregnant women, it is also important to assess which aspects of sleep are disturbed. Therefore, focusing on two different pathologies of “severity” and “impact” enables more appropriate interventions for the ISI to be used as a screening tool for insomnia among pregnant women. The “severity” focuses on the degree of sleep disturbance; if it is high, it is necessary to focus on each item more carefully and consider intervention using insomnia features. Given the noteworthy sleep characteristics of pregnant women, difficulty falling asleep and awakenings during the night are common phenomena, even in non-morbid cases. As difficulty falling asleep and awakening in the middle of the night are included in the “severity” factor, it is possible that pregnant women have higher average scores on this factor compared with the general adult population, even though their scores are normal. Therefore, when assessing sleep disorders in pregnant women, the “impact” rather than the “severity” may be more helpful in ascertaining the morbidity. Regarding “impact”, pregnant women may experience difficulties in daily life because of insomnia. Therefore, attention should be paid to their mental health, including complaints. Insomnia during pregnancy is associated not only with adverse events, but also with worsening mental health, leading to depression and suicidality [21]. Furthermore, its effects continue until postpartum, having long-term impacts [15]. Insomnia, depression, nausea, and vomiting can explain a significant portion of women’s disorders in early pregnancy [38]; nausea, vomiting, and depression during pregnancy are originally strongly related to sleep quality. Sleep-related mental health conditions and physical symptoms are also included in the care regimens of pregnant women. What remains to be studied are the causal associations of insomnia with other mental health issues such as depression, suicidality, emesis, and fear of childbirth. This topic may be approached using cross-lagged models in a prospective follow-up design. Moreover, the identification of a discrete category of insomnia during pregnancy may be important. This may be approached using cluster analyses and taximetrics. 

In this study, configural invariance, measurement invariance, and factor invariance were identified, in addition to the ISI factor structure in Japanese pregnant women, which is a new finding. In this study, we statistically tested whether the measurement invariance of the ISI could be ensured regardless of the characteristics of the pregnant women (primipara vs. multipara) and the timing (gestational weeks 10 vs. 13 weeks). Constructive invariance was confirmed, meaning that each group was found to have the same pattern of indicators and factors. The confirmed metrological invariance meant that the factor loadings of similar indicators were confirmed to be invariant across groups. The confirmed scalar invariance meant that the intercepts of similar items were confirmed to be invariant across groups. The confirmed residual invariance meant that residuals of similar items were confirmed to be invariant across groups. The confirmed factor variance invariance meant that the variance of similar factors was invariant across groups. The factor covariance invariance was confirmed, which meant that the covariance among factors was confirmed to be invariant across groups. The confirmed factor mean invariance meant that the factor was confirmed to be invariant across groups. The measurement invariance of this study supports the use of the ISI with pregnant women as a scale that can be used with the “severity” and “impact” two-factor structure reliability.

There are some limitations of this study. This study confirmed the measurement invariance of the ISI for pregnant women; however, the factor structure may be different for other subjects. In previous studies, different factor structures have been obtained for different subjects, with some studies using two factors [22,25,27] and others using three factors [20,23] or one factor [26], and the item structures of their subscales also differed. In addition, many previous studies only had factor structures with either an EFA or CFA [36]. To ensure the reliability of a scale, we believe that only after conducting an EFA and CFA and confirming the measurement invariance and structural invariance can be an appropriate scale to be used for a given subject. Therefore, as a suggestion for future research, the ISI may have a different factor structure depending on the subject, and it is necessary to confirm the measurement invariance of the ISI scale for a specific subject. In addition, the participants were limited to mothers whose gestational periods were between 10 and 13 weeks, and we used a medium-sized convenient sample. Further research is required to confirm the factor structures in larger sample sizes and participants with different gestational periods.

## 5. Conclusions

The ISI is a convenient screening tool for insomnia in pregnant women as a two-factor structure, with measurement and structural invariance across perinatal measurement time points. Focusing not only on total scores and cutoff points but also on the two subscale items of “severity” and “impact” may allow for more appropriate interventions for sleep disturbances in pregnant women.

## Figures and Tables

**Table 1:** Mean, *SD*, skewness, and kurtosis values of the ISI items (*n* = 382).

Item No.	*n*	Contents	Mean (*SD*)	Skewness	Kurtosis
1	378	Severity of sleep onset	1.9 (1.0)	0.80	−0.27
2	379	Sleep maintenance	2.2 (1.1)	0.40	−0.78
3	378	Early morning awakening problems	2.0 (1.1)	0.80	−0.33
4	379	Sleep satisfaction	3.1 (0.9)	−0.22	0.03
5	379	Interference of sleep difficulties with daytime functioning	2.2 (1.0)	0.45	−0.40
6	372	Noticeability of sleep problems by others	1.9 (1.0)	0.73	−0.31
7	379	Distress caused by the sleep difficulties	1.8 (0.9)	0.82	−0.20

**Table 2:** The EFA of the ISI (*n* =184).

Item	Communality	1-Factor	2-Factor		3-Factor		
		I	I	II	I	II	III
1	0.49	**0.70**	0.26	**0.56**	0.22	**0.50**	0.13
2	0.39	**0.62**	−0.08	**0.93**	−0.05	**0.91**	0.01
3	0.30	**0.54**	−0.06	**0.78**	−0.02	**0.79**	−0.06
4	0.43	**0.65**	**0.36**	**0.38**	−0.02	0.00	**0.94**
5	0.69	**0.83**	**0.83**	0.03	**0.78**	0.01	0.09
6	0.59	**0.77**	**0.84**	−0.04	**0.91**	0.03	−0.15
7	0.63	**0.80**	**0.87**	−0.04	**0.82**	−0.05	0.08

Factor loading > 0.3 in bold.

**Table 3:** Measurement and structural invariance.

	*χ* ^2^	*df*	*χ* ^2^ */df*	Δ*χ*^2^*(df)*	CFI	ΔCFI	RMSEA	ΔRMSEA	Judgement
Primiparas (*n* = 168) vs. multiparas (*n* = 210)									
Configural	76.948	26	2.960	Ref	0.958	Ref	0.072	Ref	Accept
Metric	83.150	31	2.682	6.202(5)	0.957	0.001	0.067	+0.005	Accept
Scalar	84.534	38	2.225	1.384(7)	0.962	+0.005	0.057	+0.010	Accept
Residual	90.016	45	2.000	5.483(7)	0.963	+0.001	0.052	+0.005	Accept
Factor variance	91.764	47	1.952	1.748(2)	0.963	0.000	0.050	+0.002	Accept
Factor covariance	91.862	48	1.914	0.098(1)	0.964	+0.001	0.049	+0.003	Accept
Time 1 (*n* = 382) vs. Time 2 (*n* = 129)									
Configural	85.441	26	3.286	Ref	0.967	Ref	0.067	Ref	Accept
Metric	93.034	31	3.001	2.701(5)	0.966	0.001	0.063	+0.004	Accept
Scalar	106.893	38	2.813	13.651(7)	0.962	0.004	0.060	+0.003	Accept
Residual	128.468	45	2.855	22.591(7)	0.954	0.008	0.060	0.000	Accept
Factor variance	128.742	47	2.739	0.324(2)	0.955	+0.001	0.058	+0.002	Accept
Factor covariance	130.616	48	2.721	0.042(1)	0.954	0.001	0.058	0.000	Accept

**Table 4:** Differences in factors’ latent means and SE values of primiparas vs. multiparas.

Factor	Factor Mean Differences	*SE*
Multipara compared with nullipara		
Severity	0.023 *^NS^*	0.087
Impact	−0.014 *^NS^*	0.075
Time 2 compared with Time 1		
Severity	−0.047 *^NS^*	0.073
Impact	0.078 *^NS^*	0.086

## Data Availability

The datasets used and analyzed in the present study are available from the corresponding author upon reasonable request.
